# Effect of pandemic influenza A virus PB1 genes of avian origin on viral RNA polymerase activity and pathogenicity

**DOI:** 10.1126/sciadv.ads5735

**Published:** 2024-12-13

**Authors:** Stephanie L. Williams, Li Qi, Zong-Mei Sheng, Yongli Xiao, Ashley Freeman, Lex Matthews, Sharon Fong Legaspi, Ervin Fodor, Jeffery K. Taubenberger

**Affiliations:** ^1^ Viral Pathogenesis and Evolution Section, Laboratory of Infectious Disease, National Institutes of Health, National Institute for Allergy and Infectious Diseases, Bethesda, MD, USA.; ^2^ Sir William Dunn School of Pathology, University of Oxford, Oxford, UK.

## Abstract

Zoonotic influenza A virus (IAV) infections pose a substantial threat to global health. The influenza RNA-dependent RNA polymerase (RdRp) comprises the PB2, PB1, and PA proteins. Of the last four pandemic IAVs, three featured avian-origin PB1 genes. Prior research linked these avian PB1 genes to increased viral fitness when reassorted with human IAV genes. This study evaluated chimeric RdRps with PB1 genes from the 1918, 1957, and 1968 pandemic IAVs in a low pathogenic avian influenza (LPAI) virus background to assess polymerase activity and pathogenicity. Substituting in the pandemic PB1 genes reduced polymerase activity, virulence, and altered lung pathology, while the native LPAI PB1 showed the highest pathogenicity and polymerase activity. The native LPAI PB1 virus caused severe pneumonia and high early viral RNA levels, correlating with elevated host cytokine signaling. Increased genetic distance from the LPAI PB1 sequence correlated with reduced polymerase activity, IFN-β expression, viral replication, and pathogenicity.

## Teaser

Past influenza pandemics contained polymerase genes from avian influenza viruses, affecting viral replication and pathogenicity.

## INTRODUCTION

Influenza A viruses (IAVs) of the family *Orthomyxoviridae* present a substantial and persistent threat to global public health, from annual human IAV, zoonotic infections, and unpredictable pandemics. Wild waterfowl, predominantly from the orders *Anseriformes* (e.g., geese, swans, and ducks) and *Charadriiformes* (e.g., gulls and shorebirds) are the main natural reservoir for IAVs. However, these wild avian IAVs can also infect a plethora of wild and domestic mammals and *Galliformes* species (*1*). Low-pathogenic avian influenza (LPAI) viruses have greatly contributed to the emergence of pandemic IAV strains in humans. It has been hypothesized that the 1918 pandemic H1N1 virus which caused approximately 50 to 100 million deaths was adapted in toto from the LPAI gene pool to humans (*2*–*4*). Two less severe pandemics subsequently occurred in 1957 and 1968. Both occurred because of the reassortment events between avian LPAI and the circulating human IAVs, which led to the emergence of the H2N2 and H3N2 pandemic strains, respectively (*5*). Thus, three of the four known IAV pandemic strains have contained a PB1 gene segment derived from the avian IAV gene pool.

The IAV has a segmented genome of negative-sense, single-stranded RNA. The eight RNA segments are individually packaged into viral ribonucleoprotein (vRNP) complexes with the IAV RNA–dependent RNA polymerase (RdRp) bound to the RNA’s 3′ and 5′ termini (*6*). The remaining RNA is encapsidated by multiple copies of IAV-encoded nucleoprotein (NP). The IAV RdRp is a heterotrimeric protein complex of the viral PB1, PB2, and PA proteins. Following viral entry and uncoating, vRNPs are transported to the host cell nucleus, where the IAV RdRp replicates and transcribes the viral genome (*7*, *8*). The PB1 protein functions as the catalytic core of the RdRp, serving an essential function in the polymerase and with implications on host adaptation, viral fitness, and pathogenicity during infection (*9*, *10*).

Because of its vital role in the RdRp, the PB1 gene is highly conserved across IAVs. The current study used a prototypical LPAI strain, A/green-winged teal/Ohio/175/1986 H2N1 (*11*). Of the pandemic PB1 genes of avian origin, the 1918 PB1 has the greatest genetic divergence to the model LPAI PB1, containing seven amino acid differences (of 757, 99% identity) (Table 1). The LPAI-derived 1957 and 1968 pandemic PB1 genes are closer in identity to the model LPAI strain PB1, with five and four respective amino acid changes. Numerous studies have described that an avian influenza PB1 gene can increase polymerase activity and RNA synthesis when reassorted into a human-adapted IAV (*12*), with further implications on overall polymerase fidelity and antigen yield in vaccine production (*9*, *13*). Furthermore, the avian IAV-origin PB1 genes from the 1968 and 1918 pandemic strains have similarly demonstrated enhanced polymerase activity, virulence, and transmissibility in vivo (*14*–*16*). However, neither the mechanisms behind these phenotypes are known, nor have the distinct pandemic avian-origin PB1 genes been evaluated experimentally in an avian IAV RdRp. Understanding how the pandemic avian-origin PB1 genes function in a LPAI RdRp could provide insight into how these PB1 genes evolved from their LPAI predecessors and allow examination of these impacts on polymerase activity and pathogenicity.

**Table 1.** Mutational comparison between the pandemic PB1 genes and model LPAI PB1.

	PB1 gene total mutation to LPAI	Synonymous mutations to LPAI	Nonsynonymous mutations to LPAI	Amino acid changes to LPAI
A/Brevig Mission/1/1918 HINI	347	340	7	K54R, N375S, E383D, V473L, L576I, V645M, S654N
A/Japan/305/1957 H2N2	164	159	5	V114I, M171I, N375S, A401V, R430K
A/Hong Kong/1/1968 H3N2	188	184	4	K121R, L212V, R327K, N375S

## RESULTS

### Phylogenetic analysis of pandemic and avian PB1 sequences

Sequence comparison was conducted to investigate nonsynonymous mutations in the pandemic and LPAI PB1 genes. The genetic distance between the 1918 PB1 and the avian H2N1 amino acid sequences was greater than that of other pandemic PB1s (Table 1). The LPAI PB1 exhibited the closest identity to the 1968 pandemic PB1, followed by the 1957 pandemic PB1. The only mutation shared between the 1918, 1957, and 1968 amino acid PB1 sequences is the N375S residue, which was previously documented (*2*). To examine how the avian PB1 genetic sequence may have changed over time, publicly available PB1 nucleotide sequences for LPAI viruses spanning 1902 through 1974 were used to generate a consensus neighbor-joining tree, using the bootstrap resampling method and no outgroup (fig. S1). The tree demonstrates that pandemic PB1 genes are in separate subclades from the 1918 PB1 (bootstrap = 87) and suggests the 1918 PB1 is genetically further in the nucleotide sequence from the model LPAI PB1 used and from the 1957 and 1968 pandemic PB1s.

### PB1 of the 1918 pandemic influenza virus reduces polymerase activity in a LPAI vRNP

**Fig. 1.**
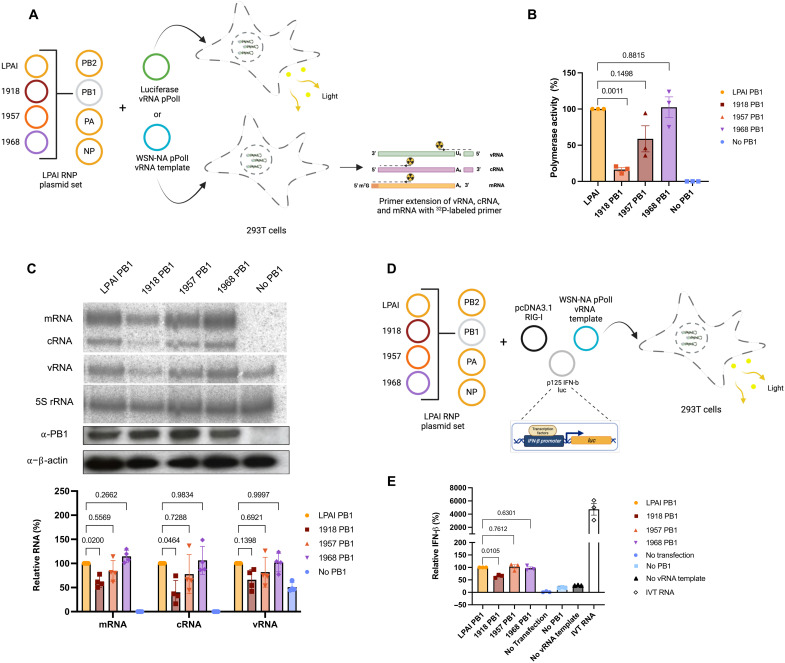
Chimeric LPAI polymerases with pandemic PB1 show reduced polymerase activity, RNA levels, and IFN-β expression. (**A**) Minigenome assays used to assess the impact of the PB1 genes of the 1918, 1957, and 1968 pandemic IAVs on the LPAI polymerase. Created in BioRender.com. (**B**) Activity of chimeric IAV polymerases in a luciferase reporter assay. Chimeric polymerase activities were presented as a percent fold compared to the activity of the LPAI polymerase. Three biological replicate ratios with SEM are shown. A one sample *t* test was done to test each ratio = 1 with Bonferroni correction. (**C**) vRNA, mRNA, and cRNA levels generated by chimeric IAV polymerases using NA vRNA template. PB1 expression levels with α-β-actin as a loading control is shown. Data are a mean of four biological replicates showing SEM. Data were normalized to the 5S rRNA loading control and expressed as a percent fold compared to the LPAI polymerase. Statistical significance was determined by two-way ANOVA and Dunnett’s multiple comparison test compared to the LPAI PB1. (**D**) Measuring IFN-β expression using a minigenome luciferase reporter gene under the control of an IFN-β promoter. Created in BioRender.com. (**E**) IFN-β levels in an IAV minigenome assay. IFN-β expression levels induced by polymerase activity were presented as a percent fold compared to the activity of the LPAI polymerase. Three biological replicate ratios with SEM are shown. A one sample *t* test was done to test each ratio = 1 with Bonferroni correction.

The effect of pandemic PB1 genes on polymerase activity in a LPAI vRNP using vRNP reconstitution assays was first investigated. The PB1, PB2_E627K_, PA, and NP proteins were expressed with a luciferase-encoding vRNA template in 293T cells, and polymerase activity was measured at 24 hours post-transfection (Fig. 1, A and B). Replacing the LPAI PB1 gene with the 1918 pandemic virus PB1 gene led to a statistically significant reduction in polymerase activity. On the other hand, substituting the LPAI PB1 gene with the 1957 and 1968 pandemic virus PB1 genes caused only a minor reduction or no change in polymerase activity.

The influenza virus polymerase transcribes vRNA into mRNA and replicates it via a complementary RNA (cRNA) intermediate. The mRNA, cRNA, and vRNA levels were measured to determine whether the observed effects on polymerase activity occurred at the transcription or genome replication stage. This was executed in a vRNP reconstitution assay using a vRNA template encoding viral neuraminidase (NA) (Fig. 1A). Replacing the LPAI PB1 with the 1918 PB1 gene significantly decreased mRNA and cRNA levels (Fig. 1C). A decrease in vRNA was also observed, although not statistically significant. Substitutions with the 1957 and 1968 pandemic PB1 genes did not significantly affect the levels of the three types of RNAs. Western blot demonstrated comparable expression of the assorted PB1 proteins, suggesting that inconsistent PB1 protein expression is not a variable in the changes in replication (Fig. 1C). These data suggest that replacing the LPAI PB1 with the 1918 PB1 gene affects transcription and replication. However, the possibility that the reduction in mRNA is the consequence of reduced replication cannot be excluded.

Influenza viruses produce RNAs that antiviral host factors can recognize. Specifically, retinoic acid–inducible gene I (RIG-I) binds influenza virus RNAs, initiating a signaling cascade that results in interferon-β (IFN-β) (*17*–*20*). A luciferase reporter assay evaluated the impact of substituting the LPAI PB1 with pandemic PB1 genes in the LPAI vRNP on INF-β expression (Fig. 1D). Substituting with the 1918 pandemic PB1 gene significantly reduced IFN-β expression (Fig. 1E). On the other hand, substitutions with the 1957 and 1968 pandemic PB1 genes had minimal to no effect on IFN-β expression. These findings suggest that IFN-β expression corresponds with the RNA levels generated by the viral polymerase.

### Chimeric LPAI viruses with pandemic PB1 genes are viable in cell culture

**Fig. 2.**
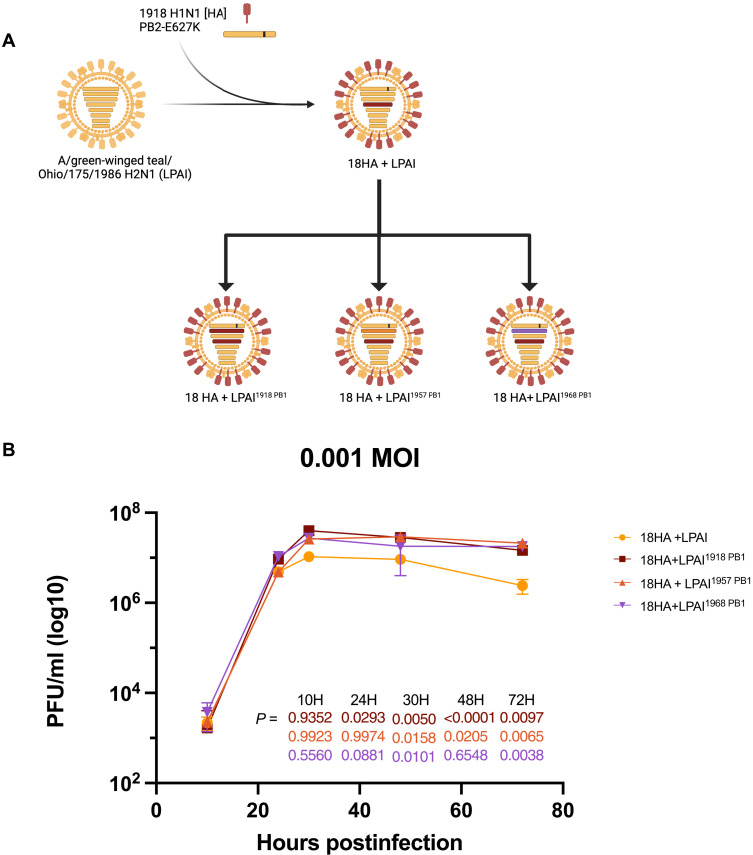
Chimeric virus rescue and corresponding growth kinetics in MDCK cells. (**A**) Recombinant viruses were generated using a LPAI prototypical virus, A/green-winged teal/Ohio/175/1986 H2N1. As previously described, the LPAI HA segment was replaced with the 1918 HA gene segment in the backbone, along with the nonsynonymous PB2 E627K mutation in PB2. The LPAI PB1 was then systematically substituted with that of the early three IAV pandemics: 1918, 1957, and 1968. Image created with BioRender.com. (**B**) Viral replication kinetics as assessed across 72 hours at an MOI of 0.001. Bars represent the SD between three technical replicates. Statistical significance was determined by two-way ANOVA and Dunnett’s multiple comparison test compared to the LPAI PB1 virus.

To examine the effects of the pandemic PB1 genes in the virus RdRp during in vitro infections, chimeric viruses were generated in which pandemic PB1 gene segments were substituted for the LPAI PB1. A previous study used the LPAI A/green-winged teal/Ohio/175/1986 H2N1 virus as a “prototypical” LPAI since each gene segment possessed a high identity to the avian IAV sequence consensus (*11*). As reported previously, the residue E627K was mutated in the LPAI PB2 gene for optimal replication in mammalian cells in vitro and in BALB/c mice without conferring a pathogenic infection (*21*). To enable the identification of alterations to mouse pathogenicity attributable to chimeric viruses encoding different PB1 genes, the LPAI model virus with a 1918 HA, previously shown to be pathogenic in mice (*11*, *21*) was used, and a set of recombinant viruses with pandemic PB1 genes was generated using reverse genetics. The 1918 HA segment, reassorted into the LPAI_PB2-E627K_ virus backbone, hereafter is referred to as the 18HA + LPAI virus (Fig. 2A). Three additional chimeric viruses carrying 1918, 1957, and 1968 pandemic PB1 gene segments were rescued through reverse genetics (*21*, *22*) and are denoted 18HA + LPAI^1918 PB1^, 18HA + LPAI^1957 PB1^, and 18HA + LPAI^1968 PB1^ viruses, respectively. All viruses and infectious samples were handled under enhanced biosafety level 3 (BSL-3+/ABSL-3+) laboratory conditions under the biosafety and biosecurity guidelines of the National Institutes of Health (NIH) Division of Occupational Health and Safety following approvals by the NIH Institutional Biosafety Committee and the NIH Dual Use Institutional Review Entity.

To address whether the replacement of the LPAI PB1 with pandemic PB1 genes affected viral replication in cell culture, their replication kinetics were tested in Madin-Darby canine kidney (MDCK) cells. Viral titers were measured over 10, 24, 30, 48, and 72 hours. All chimeric viruses replicated well in cell culture (Fig. 2B). It was noted that the 18 HA + LPAI virus had lower titers than the pandemic PB1 viruses, especially at later time points. However, as the titer differences were less than 1-log, the biological significance of this observation remains unknown.

### Chimeric LPAI viruses with pandemic PB1 genes show variable attenuation of murine pathogenicity in vivo

**Fig. 3.**
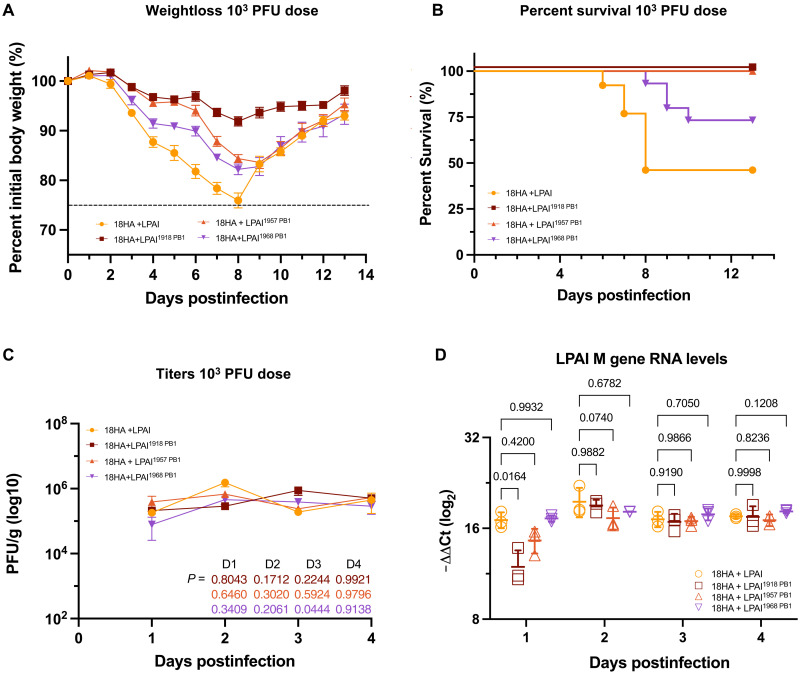
Virulence and replication of chimeric 18HA + LPAI viruses in BALB/c mice. (**A**) Weight loss of infected BALB/c mice across 13 days at the 10^3^ PFU dose in three separate experiments where groups of five mice were infected per virus (*n* = 15). SEM of data shown. A dashed line represents the 75% initial body ethical endpoint for euthanasia. (**B**) Kaplan-Meier survival curve demonstrating percent survival of mice following infection with rescued viruses at the 10^3^ PFU dose. (**C**) Viral titers of chimeric 18 HA + LPAI viruses postinfection from day 1 through day 4 (*n* = 3 per virus per day, except for 1957 day 4, which was *n* = 2 due to sample contamination). Bars represent the SD between three technical replicates. Statistics were completed via two-way ANOVA, mixed-effect analysis with Dunnett test for multiple comparisons compared to the LPAI PB1 virus. (**D**) Total viral RNA levels quantified across days 1 through 4 postinfection. Bars represent the SD between three technical replicates. Statistics were completed via two-way ANOVA, mixed-effect analysis with Dunnett test for multiple comparisons compared to the LPAI PB1 virus.

Since the LPAI chimeric viruses with the pandemic PB1 gene segments replicated comparably in a mammalian cell system, the pandemic PB1’s influence on replication and pathogenicity in vivo was evaluated in mice. A cohort of 7- to 8-week-old female BALB/c mice was intranasally infected with each respective virus at 10^2^, 10^3^, and 10^4^ plaque-forming unit (PFU) doses to ascertain the median lethal dose (LD_50_). Two additional experiments at the 10^3^ PFU dose were completed for each virus. Daily weights were taken for 13 days following infection. As determined by NIH Animal Safety Protocol (ASP# LID6E), mice were humanely euthanized when 25% of their initial body weight was lost during the experiment. The LD_50_ for each virus was calculated (table S1 and fig. S2). The 18HA + LPAI virus demonstrated high pathogenicity, while infections with chimeric viruses possessing pandemic PB1 genes led to varying declines in pathogenicity. The 18HA + LPAI^1918 PB1^ virus led to the most attenuated infections in vivo, yielding an LD_50_ > 10^4^. Mice infected at the 10^3^ PFU dose with the 18HA + LPAI^1918 PB1^ virus did not lose more than 10% of their initial body weight when observed for 13 days postinfection (Fig. 3A). The 18HA + LPAI^1957 PB1^ and 18HA + LPAI^1968 PB1^ viruses generated more pathogenic infections than the 18HA + LPAI^1918 PB1^ virus. However, 100% of mice infected with the 18HA + LPAI^1957 PB1^ virus recovered from 10^3^ PFU dose infection. Mice infected with the 18HA + LPAI^1968 PB1^ virus lost comparable weight to those infected with the 18HA + LPAI^1957 PB1^ virus but yielded a 73.3% survival rate at the 10^3^ PFU dose (Fig. 3B and table S1).

### LPAI PB1 leads to higher viral RNA levels in the early stages of viral infection

As the virus with the LPAI PB1 in the cognate RdRp led to higher levels of pathogenicity compared to the pandemic PB1-containing viruses, the next experiments examined differences in viral replication during early in vivo infection. Mice were infected at the 10^3^ PFU dose to examine the effect the PB1 substitutions had on the propagation of infectious virus in vivo. Lungs were harvested during days 1 to 4 postinfection, and the homogenate was used to quantify viral titers and measure the viral RNA (Fig. 3, C and D). No significant differences in viral titers were observed (Fig. 3C).

Total RNA was extracted from lung homogenate to quantitate virus RNA accumulated during days 1 to 4 postinfection via reverse transcription quantitative polymerase chain reaction (RT-qPCR) of the LPAI M1 gene (Fig. 3D). The dCt values for each virus were calculated and normalized to the mock-infected negative control mice. The 18HA + LPAI virus generated significantly higher viral RNA levels at day 1 postinfection than the 18HA + LPAI^1918 PB1^ virus. However, there were no significant differences in virus RNA levels between the 18HA + LPAI virus and the pandemic PB1 viruses on days 2 to 4 postinfection, suggesting that early replication could be a contributing factor to reductions in viral pathogenesis attributed to the pandemic PB1 gene segments in the LPAI RdRp.

The host immune response can be activated during the replication and transcription of the IAV genome (*23*). RNA was extracted from lung homogenate to elucidate the host gene expression levels of the cytokines and chemokines IFN-β, tumor necrosis factor–α (TNF-α), C-C motif ligand 2 (CCL2), and interleukin-1β (IL-1β) (fig. S3). Mice infected with the 18HA + LPAI virus induced higher IFN-β expression up to 2 days postinfection than those infected with viruses with the pandemic PB1 genes. Infection with the 18HA + LPAI^1957 PB1^ virus yielded the lowest IFN-β expression across these two time points (fig. S3A). IFN-β expression rose for the pandemic-PB1 viruses at days 3 and 4 postinfection to levels comparable to 18HA + LPAI virus infection. When infected with the 18HA + LPAI virus, the expression for TNF-α, CCL2, and IL-1β was elevated at day 2 postinfection (fig. S3, B to D). Expression levels of TNF-α, CCL2, and IL-1β for pandemic PB1-containing viruses rose on days 3 and 4 postinfection.

### The pandemic PB1 viruses showed attenuation in pathogenicity as compared to the 18HA + LPAI virus

**Fig. 4.**
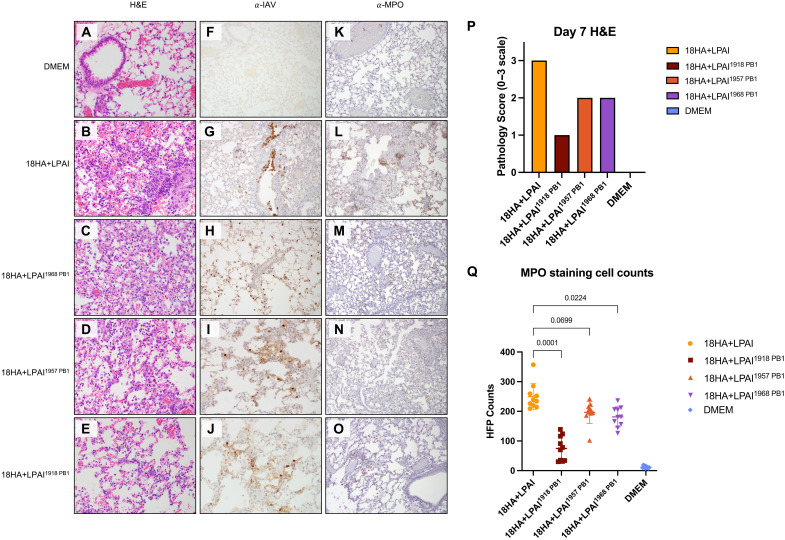
Pathology and IHC influenza A and MPO staining of mouse lung tissue. IAV and MPO antibody staining of BALB/c mouse lung at day 7 postinfection of LPAI model virus with respective pandemic PB1 genes. (**A**) DMEM negative-control mock-infected mouse lung with H&E staining. (**B** to **E**) H&E staining for chimeric 18HA + LPAI viruses. (**F** to **J**) α-IAV staining of day 7 lung histopathology postinfection with chimeric PB1 genes and DMEM mock. (**K** to **O**) α-MPO staining of day 7 lung histopathology. (**P**) Lung pathology scores counted for two replicates of each infection group evaluated on a 0 to 3 scale. Zero indicated no pathology, and 3 represented severe pathology and the highest tissue involvement. (**Q**) Cell counting of MPO staining across 10 frames. Bars represent SD. Statistics were done in Prism 9 with repeated-measures, one-way ANOVA and Dunnett multiple comparison test to the LPAI PB1 virus.

To evaluate how pathogenicity may have been altered near the weight loss nadir following infection with the chimeric viruses, lungs were harvested at the 10^3^ PFU dose on day 7 postinfection for formalin fixation to examine histopathology via hematoxylin and eosinophil (H&E) staining and immunohistochemistry (IHC; Fig. 4, A to O). A lung pathology scoring system of 0 to 3 (where 0 represented no pathology, and 3 represented severe pathology and the greatest percentage of lung tissue involvement). The average score was derived from two replicate mice per group evaluated blindly by pathologist J.K.T. (Fig. 4P), as previously described (*21*). Mock-infected mouse lung showed no abnormal changes, with a pathology score 0 (Fig. 4A). H&E staining of mouse lung sections revealed a variable pneumonia range in all virus infections (Fig. 4, B to E). In line with the weight loss data, infection with the 18HA + LPAI virus caused severe pneumonia at day 7 postinfection with prominent, widespread viral pneumonia with acute alveolitis and high accumulation of immune cells in the alveolar interstitial spaces and alveolar airspaces, with a pathology score of 3 (Fig. 4, B and P). The 18HA + LPAI^1968 PB1^ lung sections were given a pathology score of 2 as this infection elicited widespread viral pneumonia but only moderate immune cell infiltration to the alveolar interstitium and airspaces (Fig. 4, C and P). Infection with the 18HA + LPAI^1957 PB1^ virus led to multifocal viral pneumonia with notably lower levels of immune cell recruitment into respiratory epithelium and airspace and was also scored with a pathology score of 2 (Fig. 4, C, D, and P). There were minimal signs of widespread pneumonia at day 7 with the 18HA + LPAI^1918 PB1^ virus in which only a low level of host macrophages and neutrophils were present in the interstitial space of the respiratory epithelium and scattered alveoli, with a pathology score of 1 (Fig. 4, E and P). This analysis demonstrated that the 18HA + LPAI infection elicited the most pathogenic phenotype in the infected mice at the 10^3^ PFU dose as compared to the pandemic PB1 viruses.

Lung sections were further examined by IHC staining. The levels and distribution of IAV antigen in the lungs were evaluated, revealing prominent, multifocal staining in all infections at day 7 postinfection (Fig. 4, F to J). Lung sections from each virus-infected mouse group displayed staining of IAV within alveolar macrophages and respiratory epithelial cells in the bronchioles and alveolar lining cells, indicating viral antigen persistence at the weight loss nadir of the infection. The levels and distribution of IAV staining in these lung sections were comparable between the 18HA + LPAI and 18HA + LPAI^1968 PB1^ viruses, while the 18HA + LPAI^1957 PB1^ and 18HA + LPAI^1918 PB1^ viruses had lower levels of IAV staining. This may indicate lower replication or enhanced viral clearance due to the host immune responses.

The lung pathology shown in Fig. 4 highlighted marked differences in immune cell infiltrates in the alveolar interstitial and airspaces, as well as in bronchiolar air spaces and submucosal regions. Neutrophils are one of the most abundant cells circulating in the bloodstream and, in animal models, are a critical component of IAV pathogenesis, as they can exacerbate tissue injury because of their pro-inflammatory response to infection (*24*–*27*). Myeloperoxidase (MPO) is a heme-presenting peroxidase most abundantly expressed by neutrophils and contributes to its antimicrobial activity (*28*, *29*). As such, IHC staining for MPO was done to quantify neutrophils in the lungs of mice infected (Fig. 4, K to O). MPO staining showed significantly higher neutrophil counts in the lungs infected with the 18HA + LPAI virus at day 7 than the 18HA + LPAI^1918 PB1^ virus (Fig. 4Q). Therefore, the marked change in pathogenicity from the 18HA + LPAI virus to the 18HA + LPAI^1918 PB1^ virus in the context of the LPAI RdRp was correlated with the lung neutrophil count. Neutrophil counts were not significantly different from those of the 18HA + LPAI^1957 PB1^ virus on day 7 postinfection, as compared to the 18HA + LPAI virus. Lung sections from 18HA + LPAI^1968 PB1^ virus infections had statistically lower neutrophil counts than lung sections from the 18HA + LPAI virus but still significantly higher than the lung sections from the 18HA + LPAI^1918 PB1^ virus infections. The reduction of lung neutrophils with the 18HA + LPAI^1968 PB1^ also correlated with the observed lower pathogenicity compared to the 18HA + LPAI virus.

## DISCUSSION

Avian IAVs have greatly contributed to the emergence of pandemic influenza. In the 20th century, three pandemic IAV strains had genes derived from the avian influenza gene pool (*2*, *5*). Prior studies have associated experimental animal pathogenicity to the PB1 of 1918 H1N1 and 1968 H3N2 pandemic viruses (*15*, *16*, *30*). The PB1 gene is highly conserved, encoding the essential IAV PB1 protein that serves as the catalytic core of the heterotrimeric RdRp (*6*, *30*). It affects the IAV polymerase’s molecular functioning and further influences viral fitness, transmission, pathogenicity, and host adaptation (*7*, *9*, *10*, *12*, *13*, *16*, *30*–*36*). Previous studies have demonstrated that the avian PB1 protein can elevate polymerase activity and RNA synthesis in human IAV polymerases (*12*, *35*, *37*). However, it has not been examined whether the ancestral avian IAV PB1s that contributed to the 1918, 1957, and 1968 pandemic strains are still able to function optimally in a model LPAI IAV RdRp following adaptation as human-adapted pandemic viruses.

This study demonstrated that chimeric viruses with pandemic PB1 genes attenuated a model LPAI in the context of avian PB2_E627K_, PA, and NP genes and reduced polymerase activity in minigenome assays in vitro when pandemic PB1 genes were expressed in a prototypical LPAI background. As the pandemic PB1s were avian IAV derived and were close in amino acid identity to the model LPAI, the variable viral attenuation and declines in polymerase activity were unexpected. This was particularly notable for the reassortant LPAI virus with the 1918 PB1, which elicited 100% survival in mice and a marked reduction in disease severity in vivo*.* Despite this marked change in infection phenotype, changes in viral titers through 4 days postinfection were biologically insignificant between chimeric viruses. However, viral titers between strains may have diverged in the late stage of infection due to viral clearance by the immune response.

There appears to be a disconnect between infectious titers and RNA levels in the data; however, former studies have found that RNA levels do not necessarily correlate with the levels of infectious virions released (*38*, *39*). Therefore, this is not an entirely unexpected observation. The reason behind this phenomenon is unknown but deserves further investigation. The 1918 PB1 had been previously correlated with enhanced replication and virulence, especially when paired with the 1918 HA and NA genes (*15*, *16*). However, these studies were in a contemporary model human IAV backbone rather than an avian IAV used here. A 2012 study further demonstrated that in single reassortment with the LPAI model PB1 gene into the reconstructed backbone of the 1918 H1N1 virus, the LPAI PB1 did not attenuate 1918 virus replication or pathogenicity (*11*).

Examination of lung histopathology further highlighted the change in virulence connected to the change in the PB1 gene. The levels of IAV antigen staining declined in notably attenuated infections such as the 1957 and 1918 PB1-containing viruses, although the IHC staining showed no marked differences in viral antigen distribution throughout the lungs between the different viral infections. This would suggest that the inflammatory response and correlating neutrophil counts as determined by MPO staining is not related to antigen distribution at day 7 postinfection. However, it may be the case that the 1918 PB1-containing virus is triggering a less pro-inflammatory response in host tissue. This would suggest that this is connected to a downstream host response to infection rather than an effect of infectious titers in tissue (*21*, *25*). This would require future, independent studies to understand the complex dynamics of differential host responses at early stages of infection.

Few nonsynonymous mutations are found between the pandemic PB1 genes and the model A/green-winged teal/Ohio/175/1986 H2N1 PB1 protein. The 1918 PB1 protein has seven nonsynonymous mutations. The 1968 and 1957 PB1 amino acid sequence identities are even closer to the LPAI PB1, with four and five nonsynonymous changes, respectively. Select 1918 PB1 mutations have been cited to influence replication, e.g., PB1 residue 473 (*36*), or are located near residues that affect pathogenicity and viral replication (*34*, *40*). Despite independently emerging from the avian IAV gene pool, the 1918, 1957, and 1968 pandemic PB1 genes share the N375S mutation (*2*). The N375S mutation is located on the base of the PB1 β-hairpin, near the PA-loop. A major biochemical change from N to S may alter the interaction between certain pandemic PB1 genes and the LPAI PA. However, the molecular implications of the PB1 375 residue mutation remain unresolved.

One caveat to address is that the model avian IAV PB1 gene used in this study is from 1986, while the pandemic PB1 that exhibits the most drastic changes in pathogenic and replication phenotypes is from 1918. Although there is a sampling deficit in avian IAVs between 1918 and the 1960s, we identified a subset of avian IAV isolates collected from 1950 to 1974 that shared high sequence identity at the nucleotide level to the 1918 PB1 gene in a distinct subclade from the other 1957 and 1968 PB1 genes. This highlights that despite a large passage of time, the PB1 genes from some more recent LPAIs in bird populations are very comparable in sequence to the 1918 PB1 (fig. S1). The deep cladal differences in the PB1 nucleotide tree suggest that synonymous changes drive the clade separation. Different clades of avian PB1 may have unknown functional incompatibilities when reassorted with the PB2 and PA genes in LPAI strains in other clades. In totality, the seven nonsynonymous mutations in the 1918 PB1 could have hindered the formation of the RdRp heterotrimer, replication dimer, or assembly of complexes with essential host proteins for replication or transcription of the viral genome.

The changes in the pandemic PB1 genes may have altered innate immune activation through changes in the activation of IFN-β or PB1-F2. Sensing of the 5′-triphosphate vRNA ends by RIG-I leads to the induction of IFN-β (*17*, *38*). Mini-vRNAs (mvRNAs) can be generated at variable levels by the different IAV polymerase from aberrant replication and were previously linked to IFN-β expression and overactivation of the innate immune response (*41*). One hypothesis could be that the viral attenuation in vivo and correlating reduced IFN-β induction early in infection may be connected to declining full-length RNA or short, aberrant mvRNA templates. It could be speculated further that, despite comparable infectious titers, the 1918 PB1-chimeric virus may be driving less pro-inflammatory response in the tissue, as noted by the lower neutrophil MPO counts at day 7 postinfection (*21*, *25*). There is also the matter of IAV accessory protein PB1-F2 in an alternate ORF of the PB1 gene (*42**–**44*). PB1-F2 is cited widely as an innate immune agonist and antagonist and a virulence factor that can have strain and host-specific effects (*43**–**47*). More research will need to be done to examine whether aberrant viral genome replication or PB1-F2 have contributing roles to the observed changes in virulence.

An avian-origin PB1 gene has arisen in three previous influenza pandemic strains (*2*, *5*). The data here demonstrated that despite the marked sequence conservation of the PB1 gene, as few as seven nonsynonymous mutations can have major downstream consequences. These changes significantly altered polymerase function with a subsequent impact on pathogenicity in an LPAI background due to hypothesized disruption of interprotein interactions in the polymerase or modulating innate immune activation. Although deleterious effects of the pandemic PB1 genes in the avian IAV polymerase were observed, these mutations may have served a role in host adaption, as these PB1s reassorted within the context of an otherwise human-adapted pandemically circulating IAV. As a result, these mutations may hold insights into the complex interprotein dynamics of an evolving IAV RdRp and emphasize the duality of the polymerase in both viral replication and pathogenicity. Understanding the molecular mechanism underpinning the function of the avian IAV PB1 and how it relates and contributes to viral pathogenesis in the context of a human IAV RdRp could provide invaluable insight into the emergence, fitness, and pathogenesis of past and future pandemic IAVs.

## METHODS

### Generation of chimeric IAVs by reverse genetics

All work with the chimeric IAVs was performed in enhanced biosafety level (BSL) 3 laboratory conditions at the NIH. Biosafety practices were aligned with NIH and the *Centers for Disease Control and Prevention Biosafety in Microbiological and Biomedical Laboratories (BMBL) 6th Edition*, the federal Select Agent program, and the NIH Guidelines for Research Involving Recombinant and Synthetic Nucleic Acids, as well as NIH policies and requirements set forth by the NIH Department of Occupational Health and Safety and NIH Institutional Biosafety Committee. This project was reviewed by the NIH Dual Use Research of Concern Institutional Review Entity periodically, and this manuscript was reviewed and approved by the IRE before submission. The IRE did not identify that this research met the definition of dual-use research of concern, nor did it determine that the research required elevation as part of the P3CO framework.

The A/green-winged teal/Ohio/175/1986 H2N1 virus, hereafter named LPAI, was donated by Dr. R. Slemons (Ohio State University, Columbus, Ohio) in a previous study (*11*, *48*). This strain was selected on account of the close identity of the genes to the consensus sequence of avian IAV sequences deposited in the National Center for Biotechnology Information (NCBI) Influenza Resource Database. The eight gene segments for LPAI were previously subcloned in the antisense into the pHH21 vector (*11*). The pHH21 plasmids for the LPAI PB2 gene was mutated in a previous study for E627K (*11*). The generation of the 1918 HA gene in pHH21 was previously described (*14*). A 1:7 reassortment virus using the A/Brevig Mission/1/1918 H1 gene in the avian LPAI_PB2-E627K_ backbone, which was used as a model virus for studying chimeric LPAI viruses with different pandemic PB1 genes has been previously described (*11*). The PB1 genes for A/Japan/305/1957 H2N2 and A/Hong Kong/1/1968 H3N2 were synthesized by GenScript (GenScript, New Jersey) and cloned into pHH21 plasmids using BsaI/BsmBI restriction digest and T4 ligation and viruses were rescued as previously described (*14*). The model 18HA + LPAI and chimeric viruses containing the PB1 gene segments of the A/Brevig Mission/1/1918 H1N1 (18HA + LPAI^1918 PB1^), A/Japan/305/1957 H2N2 (18HA + LPAI^1957 PB1^), and A/Hong Kong/1/1968 H3N2 (18HA + LPAI^1968 PB1^) were rescued as previously described (*22*). The rescued viruses were cultured and quantified by plaque assay. Stock vRNA was deep sequenced for sequence confirmation.

### Viral growth kinetics in MDCK cells

MDCK [American Type Culture Collection (ATCC), CCL-34] cell culture was grown to 90% confluency in six-well plates in Dulbecco’s Modified Eagle Medium (DMEM) + 10% fetal bovine serum before infection. Viruses were diluted in DMEM for infection at a 0.001 multiplicity of infection (MOI) with three technical replicates. Virus titers were measured 10, 24, 30, 48, and 72 hours postinfection via plaque assay.

### In vivo mouse studies

Animal studies using chimeric LPAI viruses were conducted in animal BSL3 (ABSL3) laboratory suites at the NIH in Bethesda, MD. Such experiments operated under the approval of an NIH Animal Care and Use Committee–approved animal study protocol (ASP) LID-6E.

BALB/c mice were obtained through JAX Mice and Services (Bar Harbor, ME), and daily animal husbandry was overseen through the NIH Building 33 Charles River contract. Under mild anesthesia of isoflurane, 7- to 8-week-old female BALB/c mice were infected intranasally at 10^4^, 10^3^, and 10^2^ PFU for each respective virus in a cage of five. Viruses were diluted in DMEM to achieve the endpoint dose. Their weights were subsequently monitored daily over 13 days postinfection. According to approved animal safety protocols, animals reaching 75% of their day 0 body weight were ethically euthanized to minimize animal suffering. An LD_50_ of each virus was calculated using the Reed and Muench method (*49*).

Viral lung titers were collected from a BALB/c mice infected at 10^3^ PFU at days 1 to 4 postinfection for each virus, including a mock-infected group. Three sets of lungs were collected per day for each virus. Lung homogenates were used to quantify viral titers and extract RNA. Titers were obtained via plaque assay. Following factory protocols, total lung RNA was extracted with TRIzol reagent (Invitrogen, USA). During processing, one of the samples for the 18 HA + LPAI^1957 PB1^ infection became contaminated, resulting in only two replicates for this virus on day 4 postinfection.

### Quantification of vRNA and gene expression via RT-qPCR

SYBR green RT-qPCR was done via the Luna Universal One-Step RT-qPCR kit (New England Biolabs no. E3005) to quantify RNA from the LPAI M1 gene and expression of IFN-β, IL-1β, CCL2, and TNF-α cytokines following factory protocols for reaction set up. The LPAI M1 gene was used as the target gene for quantification. Expression of mouse β-actin housekeeping gene was used as a control (*50*). The primers for targeted genes are described in table S2. The extracted RNA was diluted 50× in nuclease-free water and 5 μl was loaded to each PCR reaction. Primers targeting LPAI M1, β-actin, IFN-β, IL-1β, CCL2, and TNF-α were used at 10 μM concentration. Applied Biosystems QuantStudio 5 Real-Time PCR System was used for running SYBR green RT-qPCR following Luna Universal One-Step RT-qPCR protocol with 45 cycles set for the denaturation and extension steps. RNA was quantified using the ddCT method with normalization to the dCT of the mock-infected mice. Host gene expression levels for IFN-β, IL-1β, CCL2, and TNF-α were analyzed using the ddCT method with normalization of the dCT to the LPAI virus–infected group.

### Minigenome assays

293T cells (ATCC, CRL-3216) were seeded the day before transfection in 96-well plates at 4.0 to 5.0 × 10^4^ cells per well to achieve 70 to 80% confluency. The plasmid expression set for the LPAI RdRp genes with the PB2 E627K mutation, and A/Brevig mission/1/1918 H1N1, A/Japan/305/1957 H2N2, and A/Hong Kong/1/1968 H3N1 PB1 previously generated (*11*, *51*, *52*). The huPolI Luc plasmid expressing firefly luciferase flanked by IAV noncoding URT on the 5′ and 3′ ends was used as a reporter for polymerase activity (*53*). Cells were transfected with the four IAV expression vectors and the luciferase plasmid at 40 ng each using TransIT-LT1 transfection reagent (Mirus Bio LLC). A transfection where PB1 was substituted by empty pCAGGs vector at 40 ng was a negative control. The cells were transfected in triplicate. Cell lysates were collected with 1× passive lysis buffer (Promega, no. E1941) 24-hours post-transfection. Luciferase reporter assays were conducted via Promega Luciferase Assay (Promega, no. E1500) factory protocol. Data presented was collected from three individual experiments. For each experiment, three technical replicates were averaged, and the average background luciferase signal was subsequently subtracted from the data. The data were then presented as a percent fold compared to the activity of the LPAI polymerase. Three biological replicates ratios were shown with standard error of the mean (SEM) and statistical significance was measured using a one sample *t* test was done to test each ratio = 1 with Bonferroni correction for three comparisons in Prism 9.

To evaluate IFN-β expression, pCAGGs plasmids encoding the LPAI RNPs and the pandemic PB1 coding sequences were transfected with a pPolI promoter plasmid encoding the WSN-NA influenza gene at 40 ng per plasmid (*11*, *51*, *52*, *54*). Lipofectamine 2000 transfection reagent (Thermo Fisher Scientific, USA) was used following factory protocols. The p125 IFN-β reporter plasmid was generously donated by J. Rehwinkel (University of Oxford, WIMM) and transfected at 40 ng in addition to a pcDNA3.1-myc–tagged RIG-I plasmid (*55*). Promega ONE-Glo Luciferase assay kit (Promega, no. E6120) was used 24 hours following transfection as per factory protocols. Data presented was collected from three individual experiments. For each experiment, three technical replicates were averaged, and the average background luciferase signal was subsequently subtracted from the data. The data were then shown as a percent fold compared to the activity of the LPAI polymerase. The SEM for the three biological replicates ratios were calculated and statistical significance was measured using a one sample *t* test was done to test each ratio = 1 with Bonferroni correction for three comparisons in Prism 9.

### Primer extension for vRNA, cRNA, and mRNA quantification

293T cells were seeded to 24-well plates 24 hours before transfection at 3.0 to 4.5 × 10^5^ cells per well to achieve 70 to 80% confluency. pCAGGs plasmids encoding the LPAI RNPs and the pandemic PB1 coding sequences were transfected with a pPolI promoter plasmid encoding the antisense WSN-NA influenza gene at 200 ng per plasmid (*11*, *51*, *52*, *54*). TRI Reagent (Sigma-Aldrich) was used to isolate total RNA following the manufacturer’s protocols 24 hours post-transfection. First-strand RNA synthesis via SuperScript III (Thermo Fisher Scientific Inc.) was conducted using NA-specific radiolabeled primers [γ-^32^P] ATP (PerkinElmer) as referenced (*54*). A primer specific to 5S rRNA was used as an internal control. Products were resolved on a 6% denaturing polyacrylamide gel electrophoresis gel with 7 M urea. The resulting gels were imaged by phosphorimaging through the FLA-5000 scanner (Fuji), and targets were quantified using Fiji ImageJ2. The data shown are the results of four independent experiments.

### Western blots

Cells were lysed using Promega lysis buffer (Promega). Factory protocols were followed for subsequent Western blotting. Primary antibodies for α-PB1 (GTX125923) from GeneTex and α-β-actin from Santa Cruz Biotechnology (sc-47778) were used. GeneTex secondary, horseradish peroxidase (HRP)–conjugated goat α-rabbit immunoglobulin G (IgG; GT213110-01) and goat α-mouse IgG (GTX213111-01) antibodies were used following probing with primary antibodies. Detection was done via Amersham ECL start Western Blotting Detection Reagent (Cytiva, RPN3243) following factory protocols.

### Histopathology

Lung samples were collected from infected mice in duplicate for each respective virus, including the DMEM-mock infected at day 7 postinfection. H&E-stained slides were prepared via Histoserv Inc. (Germantown, MD). Unstained sections on charged slides were generated for α-IAV and α-MPO immunohistochemical staining. The primary antibody used for α-IAV staining was acquired from Abcam (ab20841) and used at a 1:200 dilution, followed by the secondary anti-goat IgG HRP (Abcam, ab6741) at 1:2000. MPO was stained with Thermo Fisher Scientific α-MPO antibody (PA5-16672) at a 1:100 dilution. Abcam secondary anti-rabbit HRP (ab205718) was used as the secondary antibody diluted to 1:2000.

### PB1 sequence alignments and phylogenetics

The sequence accession numbers of the pandemic PB1 and model LPAI PB1 used in the experiments are as follows: A/Brevig Mission/1/1918 H1N1 (ABA55039.1), A/Japan/305/1957 H2N2 (ACU79967.1), A/Hong Kong/1/1968 H3N2 (AAK51714.1), and A/green-winged teal/Ohio/175/1986 H2N1 (AMB21957.1). The nucleotide and amino acid sequences of the PB1 genes already exist in the public domain and were retrieved from the Influenza Research Database (IRD; 2021). A global alignment was conducted in Geneious Prime 9 (2020). Unique PB1 nucleotide sequences for IAVs from 1902 to 1974 were downloaded from the NCBI Influenza Resource Database. Select North American Turkey H1N1 isolates were removed because of potential misidentification as classical swine H1N1 (*56*, *57*). An unrooted, consensus neighbor-joining tree was generated from the remaining nucleotide sequences with the Geneious Prime tree builder with the Tamura-Nei genetic distancing model with no outgroup and calculated bootstrap values.

### Quantification and statistics

All statistics were conducted on Prism 9, with the statistical tests described in Methods and figure legends. Significance was noted for *P* values < 0.05. Statistics were done in consultation with statisticians at the NIH. Graphs were generated using Prism 9.
