# Supplementary material for: Effect of pandemic influenza A virus PB1 genes of avian origin on viral RNA polymerase activity and pathogenicity
Source: Sci Adv. 2024 Dec 13;10(50):eads5735. doi: 10.1126/sciadv.ads5735 (PMC11641000; doi:10.1126/sciadv.ads5735)
Supplement: Supplementary file 1 — Figs. S1 to S3 Tables S1 and S2 [file sciadv.ads5735_sm.pdf]

Science Advances
AAAS

Supplementary Materials for

Effect of pandemic influenza A virus PB1 genes of avian origin on viral RNA polymerase activity and pathogenicity

Stephanie L. Williams *et al.*

Corresponding author: Jeffery K. Taubenberger, taubenbergerj@niaid.nih.gov

Sci. Adv. **10**, eads5735 (2024)
DOI: 10.1126/sciadv.ads5735

This PDF file includes:

Figs. S1 to S3
Tables S1 and S2

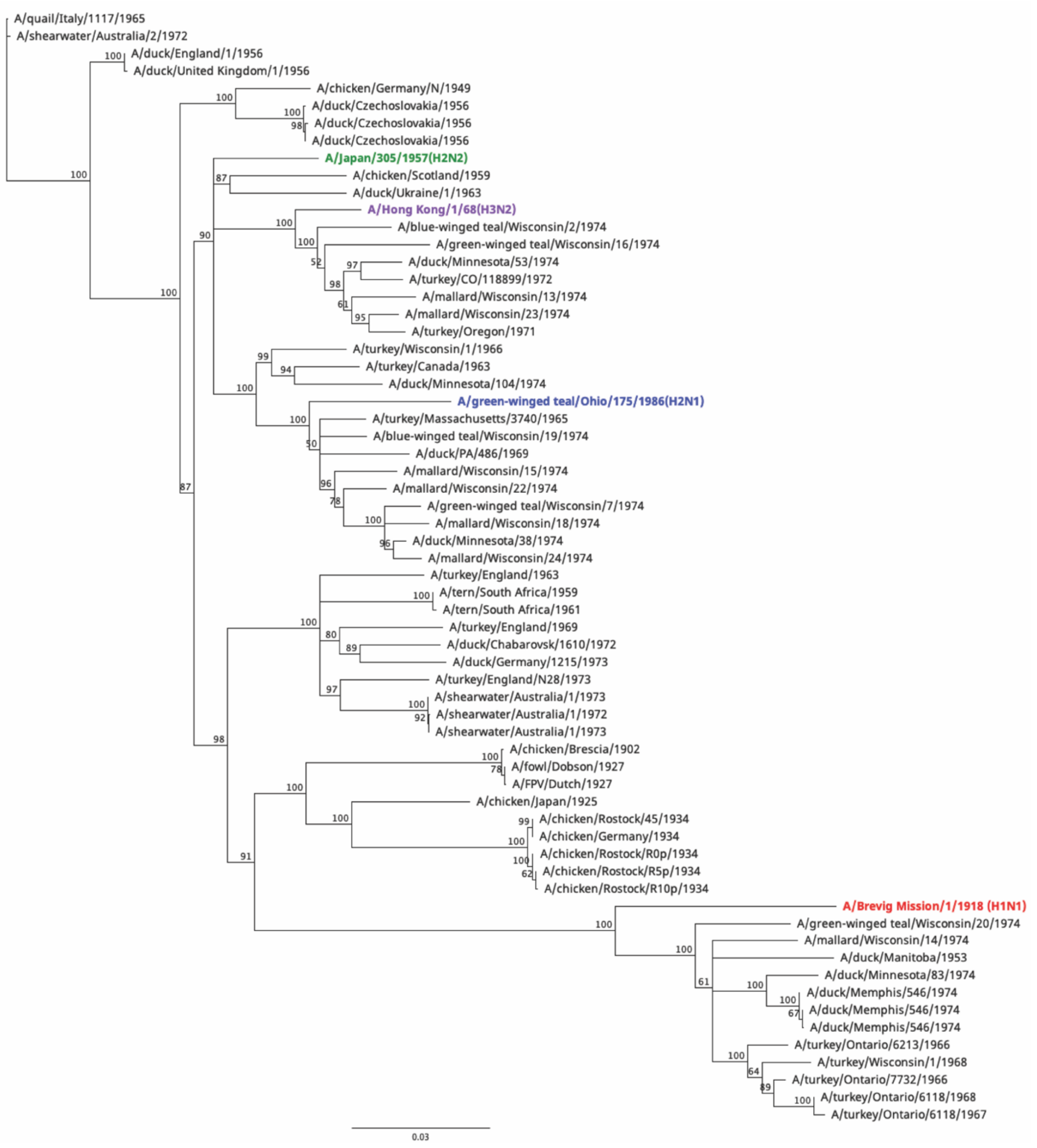
A/quail/Italy/1117/1965
A/shearwater/Australia/2/1972
A/duck/England/1/1956
A/duck/United Kingdom/1/1956
A/chicken/Germany/N/1949
A/duck/Czechoslovakia/1956
A/duck/Czechoslovakia/1956
A/duck/Czechoslovakia/1956
A/Japan/305/1957(H2N2)
A/chicken/Scotland/1959
A/duck/Ukraine/1/1963
A/Hong Kong/1/68(H3N2)
A/blue-winged teal/Wisconsin/2/1974
A/green-winged teal/Wisconsin/16/1974
A/duck/Minnesota/53/1974
A/turkey/CO/118899/1972
A/mallard/Wisconsin/13/1974
A/mallard/Wisconsin/23/1974
A/turkey/Oregon/1971
A/turkey/Wisconsin/1/1966
A/turkey/Canada/1963
A/duck/Minnesota/104/1974
A/green-winged teal/Ohio/175/1986(H2N1)
A/turkey/Massachusetts/3740/1965
A/blue-winged teal/Wisconsin/19/1974
A/duck/PA/486/1969
A/mallard/Wisconsin/15/1974
A/mallard/Wisconsin/22/1974
A/green-winged teal/Wisconsin/7/1974
A/mallard/Wisconsin/18/1974
A/duck/Minnesota/38/1974
A/mallard/Wisconsin/24/1974
A/turkey/England/1963
A/tern/South Africa/1959
A/tern/South Africa/1961
A/turkey/England/1969
A/duck/Chabarovsk/1610/1972
A/duck/Germany/1215/1973
A/turkey/England/N28/1973
A/shearwater/Australia/1/1973
A/shearwater/Australia/1/1972
A/shearwater/Australia/1/1973
A/chicken/Brescia/1902
A/fowl/Dobson/1927
A/FPV/Dutch/1927
A/chicken/Japan/1925
A/chicken/Rostock/45/1934
A/chicken/Germany/1934
A/chicken/Rostock/R0p/1934
A/chicken/Rostock/R5p/1934
A/chicken/Rostock/R10p/1934
A/Brevig Mission/1/1918 (H1N1)
A/green-winged teal/Wisconsin/20/1974
A/mallard/Wisconsin/14/1974
A/duck/Manitoba/1953
A/duck/Minnesota/83/1974
A/duck/Memphis/546/1974
A/duck/Memphis/546/1974
A/duck/Memphis/546/1974
A/turkey/Ontario/6213/1966
A/turkey/Wisconsin/1/1968
A/turkey/Ontario/7732/1966
A/turkey/Ontario/6118/1968
A/turkey/Ontario/6118/1967
0.03

Fig. S1.

Phylogenetic tree of LPAI nucleotide sequences from 1902-1974 with pandemic PB1 genes and A/green-winged teal/Ohio/175/1986 H2N1. Unrooted tree of nucleotide sequences from isolates spanning from 1902 through 1974 mapped with the pandemic PB1 gene sequences from 1918, 1957, 1968, and the LPAI model PB1, A/green-winged teal/Ohio/175/1986 H2N1. Neighbor-joining consensus tree generated in Geneious Prime using the Geneious Tree Builder with the Tamura-Nei genetic distancing model with no outgroup. Bootstrap values from 100 replicates are labeled on branches.

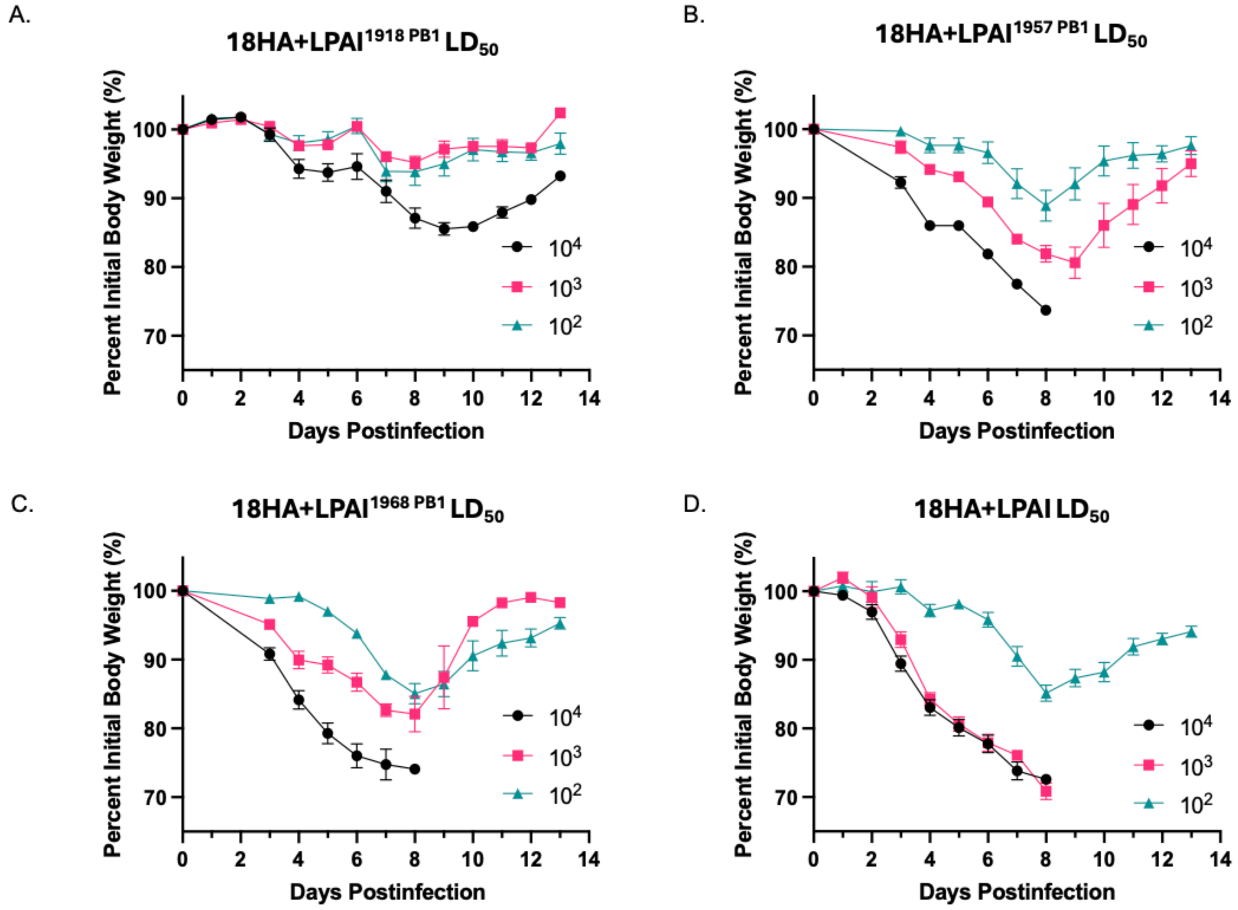

Fig. S2.

LD_{50} weight loss graphs for chimeric viruses. **A-D)** Each graph is indicative of the PB1 gene in the 18HA+LPAI backbone. Data is the result of a single experiment where mouse cohorts were infected in groups of 5 for each virus, at each respective dose.

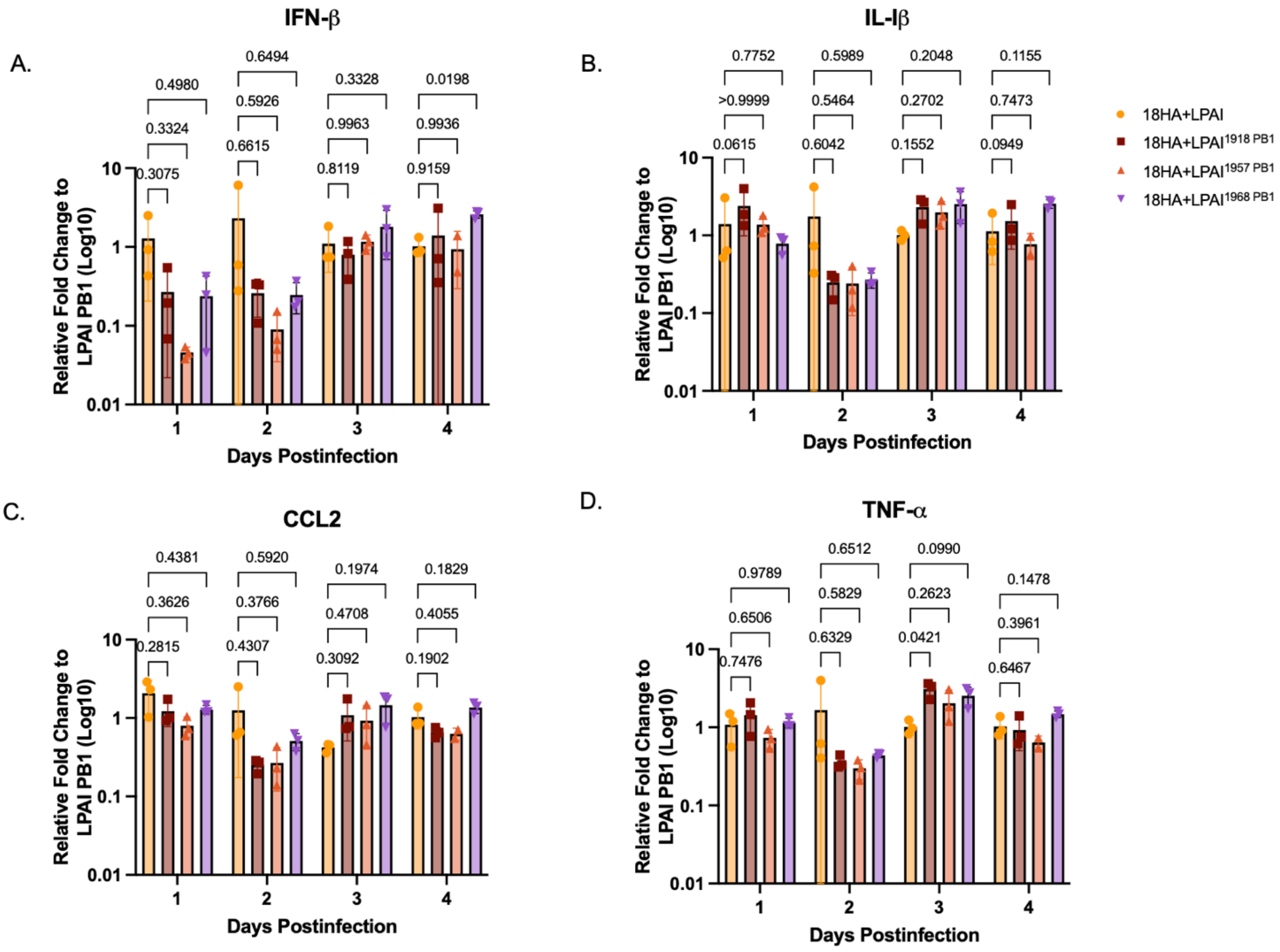

Fig. S3.

RT-qPCR data of IFN-β (A), IL-1β (B), TNF-α (C), and CCL2 (D) expression at days 1 through 4 post-infection. Total RNA was extracted from lung homogenate (n=3 per virus per day, except for 1957 day 4, which was n=2 due to sample contamination). ddCT was used to quantify cytokine expression, and the dCT value was normalized for the LPAI virus-infected group. Bars

represent standard deviation. Two-way ANOVA, a mixed-effect model with the Dunnett test for multiple comparisons compared to the LPAI PB1 virus, was conducted in Prism 9.

Table S1.
Percent survival 10^3 (n=15) and LD_{50} in BALB/c mice

PB1 in 18HA+LPAI virus	*Infected BALB/c Mice (n)*	*Number of mice survived*	*Percent Survival*	*50% Lethal Dose (LD_{50})*
LPAI PB1	15	6	40%	$10^{2.5}$
1918 PB1	15	15	100%	$>10^4$
1957 PB1	15	15	100%	$10^{3.5}$
1968 PB1	15	11	73.3%	$10^{3.1}$

Table S2.

RT-qPCR primer names and sequences

Name	*Primer pairs (5'→3')*
mβAct	GCACGTTGACATCCGTAAAG<br>GAGGAGCAATGATCTGAAG
LPAI M	ATGGTGACAACAACCAACCC<br>GCACTGGAGCTAGGATGAGTCCCA
mIFN-β	CCTACAGGGCGGACTTCAAG<br>TCTTGGATCCGAAAGGCAGT
mIL-1β	TGGACCTTCCAGGATGAGGACA<br>GTTCAGCGCGGAGCCTGTAGTG
mTNF-α	GGTGCCTATGTCTCAGCCTCTT<br>CACTCCAGCTGCTCCTCCAG
mCCL2	GCTACAAGAGGATCACCAGCAG<br>GTTGTAGGTTCTGATCTCATTTGGTTC
